# Use and Abuse of Drug-Habits

**Published:** 1889-08

**Authors:** 


### Editorial.

### USE AND ABUSE OF DRUG-HABITS.

To the already long list of drugs, the use of which, under proper restrictions, is both beneficial and proper in combating the various ills to which flesh is heir, but whose abuse becomes a curse to humanity, another has recently been added. Scarcely have we learned to properly use antipyrin than the tocsin of alarm must be sounded against its abuse. The recent discovery of its value as a nerve-tonic places it on the list with morphine, chloral, cocaine, etc., so seductive is its gentle soothing influence upon the over-strained nerves.

Its victims are already found, especially among society women, whose nerves, strung up to a high pitch by the overwhelming demands of a winter season of gayety, seize eagerly upon anything that will afford relief from the headaches and other disorders arising from prolonged fatigue and overtried nerves. So pleasing is the effect that it is soon used for every trifling ill feeling, until the patient finds herself unable to live without it, and the fascinating "antipyrin-habit" is formed.

Properly used as a nerve-tonic, its effects are admirable, but *abused*, the victim becomes even more hopelessly entangled than the morphine or the cocaine victim.

The effects vary with the dose. In large doses it produces complete relaxation with loss of reflex action. In moderate doses, continued, it induces convulsions. As a stimulant its effect is much like that of quinine.

